# Assessment of Genetic Diversity of a Collection of *Senna obtusifolia* (L.) Irwin and Barneby Using SSRs Markers in Burkina Faso

**DOI:** 10.1155/2023/3761799

**Published:** 2023-06-05

**Authors:** Haoua Nacambo, Nerbéwendé Sawadogo, Teyiouê Benoit Joseph Batieno, Mahamadi Hamed Ouedraogo, Abdoul Kader Congo, Wend-Pagnagde Felicien Marie Serge Zida, Kiswendsida Romaric Nanema, Mahamadou Sawadogo

**Affiliations:** ^1^Equipe Génétique et Amélioration des plantes, Laboratoire Biosciences, Université Joseph KI-ZERBO, 03 BP 7021 Ouagadougou 03, Burkina Faso; ^2^Institut de l'Environnement et de Recherche Agricole (INERA), CNRST, 01 BP 476 Ouagadougou 01, Burkina Faso

## Abstract

*Sennaobtusifolia* (L.) is a plant in the genus *Senna* that contributes to improving nutritional quality, food security, and better health protection for rural populations. However, very few studies have been devoted to it in Burkina Faso. Consequently, its genetic diversity remains poorly known. Such neglect would lead to the erosion of its genetic resource. The general objective of this study is to contribute to a better knowledge of the genetic diversity of the species in order to be able to issue scientific bases for its conservation, valorization, and genetic improvement. Sixty (60) accessions of *Senna obtusifolia* were collected in the wild from five provinces of three climatic zones of Burkina Faso. Molecular characterization was carried out using 18 SSR markers. Fifteen were polymorphic microsatellite markers leading one hundred and one (101) alleles in total, with an average of seven (7) alleles per locus. The number of effective alleles was 2.33. Expected heterozygosity, Shannon diversity index, and polymorphism information content averaged 0.47, 1.05, and 0.47. Molecular characterization revealed the existence of genetic diversity within the collection. This diversity has been structured into three genetic groups. Genetic group 3 presents the highest genetic diversity parameters.

## 1. Introduction

*Senna obtusifolia* (L.), also known as Foetid Cassia in French or *Katr-nanguri* in Mooré, is an annual or perennial herb with an erect, branched stem that can reach an average height of 2.5 m and has alternate, pinnate leaves [1, 2]. According to Irwin and Barneby [3], *Senna obtusifolia* occurs in two forms: one that originates from the Caribbean which has an extrafloral uniglandular nectary on the upper surface of the rachis located between the two lower leaflets and with a chromosomal formula of 2*n* = 28. The diameter of the pods varies between 3.5 and 6 mm and the other originates from British Guiana, Surinam, and Venezuela which has two extrafloral nectaries. Its chromosomal formula is 2*n* = 26, and its pods are relatively narrower with a diameter varying between 2 and 3.5 mm.

It is a plant that contributes to the improvement of nutritional quality, food security, and better health protection of rural populations [4]. Indeed, its leaves are used as a vegetable in Africa and Asia [5, 6]. In addition, according to the chemical composition reported by Busson [7], the plant contains more iron (6 mg), calcium (608 mg), phosphorus (95 mg), vitamin C (120 mg), and protein (5.66 mg) than *Amaranthus hybridus* and *Hibiscus sabdariffa* and more vitamin A than *Hibiscus sabdariffa* per 100 grams of fresh matter, which, however, are already domesticated and widely consumed species. High levels of iron and vitamins A and C represent particularly important health issues in countries plagued by numerous cases of anemia caused mainly by malaria [8]. The leaves, seeds, and roots of *Senna obtusifolia* (L.) are used to cure diseases such as night blindness, hepatitis, ulcers, stomach ulcers (leaves), skin diseases, conjunctivitis, and poisonous insect bites (seeds); diseases caused by helminths, against herpes and the expulsion of intestinal worms (roots) [9]. However, very few studies have been devoted to it in Burkina Faso, and its diversity remains poorly known. Several studies have been conducted mainly on their medicinal role [9]; biochemical composition [10]; ethnobotanical study [4]; agromorphological characterization [11], and competitiveness evaluation of it as an invasive species [12].

Agromorphological characterization of 70 accessions including the present collection of *Senna obtusifolia* was conducted in 2018 by Nacambo et al. [11] using morphological markers. This study showed the existence of variability within the *Senna obtusifolia*. The morphological markers used are the number of days to emergence, number of days to flowering, number of primary branches, leaf biomass, length of the fruit, fruit width, number of fruits per plant, number of seeds per fruit, and number of seeds per plant.

According to Banerjee et al. [13], agromorphological markers although indispensable for the evaluation of variability are often under the influence of the environment, thus do not allow a better appreciation of genetic variability. Thus, since 1980, molecular markers that offer a better approach to diversity have been developed [14]. Knowledge of the intraspecific variability of a plant is important in the process of conservation and development of its genetic resources [15]. It is therefore necessary to better appreciate the variability observed during the agromorphological evaluation using molecular markers. Work carried out by Mohanty et al. [16] revealed a total of 158 alleles, 109 alleles, and 78 alleles for seventeen (17) RAPD markers, eleven (11) ISSR markers, and seven (7) SSR markers on *Senna obtusifolia* (*Cassia tora*).

Many molecular markers exist; however, simple sequence repeat (SSR) markers are excellent tools for cultivar identification, pedigree analysis, and assessment of genetic distances within many plant species. They are also excellent tools for the assessment of genetic diversity because of their codominant nature and their ability to be analyzed with quality [17, 18]. As molecular markers, SSRs combine many properties desirable for markers, including high levels of polymorphism and information content and unambiguous designation of alleles [19]. Indeed, according to Sorrells [20], they are considered a class of markers that contribute to the direct selection of alleles. This is how this activity comes into play, which aims at a better knowledge of the genetic diversity within a collection of the species on the basis of SSR markers. The specific objectives were (i) to determine the level of genetic diversity within the collection and (ii) to determine the population structure within the collection of *Senna obtusifolia*.

## 2. Material and Methods

### 2.1. Material

#### 2.1.1. Plant Material

**Figure 1:**
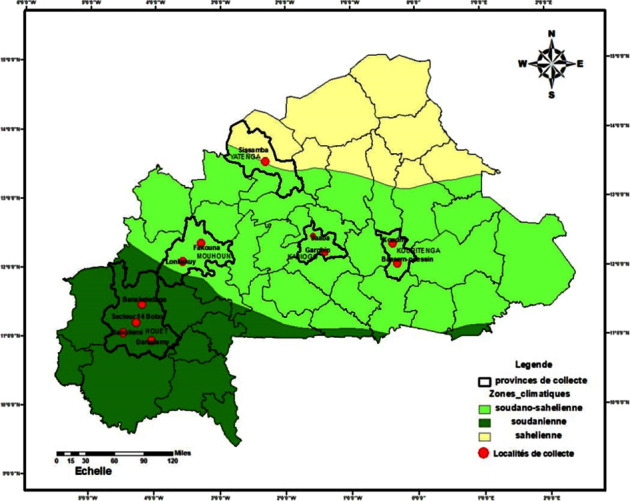
Location of the sites of prospection and collection of the accessions of *Senna obtusifolia* (L.).

**Figure 2:**
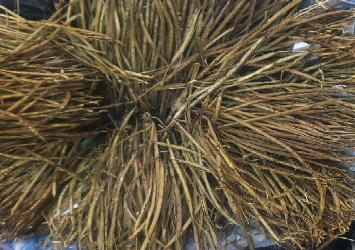
Accessions of *Senna obtusifolia* collected.

The plant material consists of sixty (60) accessions of *Senna obtusifolia* (L.) collected during a survey. They come from ten (10) villages distributed in five (5) provinces of the three climatic zones surveyed and sector 14 of the city of Bobo-Dioulasso (Figure 1). These included 9 (15%) accessions from the Sahelian zone, 33 (55%) from the Sudano-Sahelian zone, and 18 (30%) from the Sudanian zone. These accessions were all collected from spontaneous populations, tied up in sachets, and stored in bags (Figure 2).

#### 2.1.2. Microsatellite Markers Used for Genetic Diversity Study

A total of eighteen (18) microsatellite markers were used in our study; they were selected from those used by Li et al. [19] on *Vigna unguiculata* (cowpea) (Table 1). These markers consist of tandem repeats of single, di, tri, or tetra nucleotide motifs and are abundant in the genome of eukaryotes [21]. These primers were used because they do not have species-specific markers. Therefore, we tested for markers of the legume family since Caesalpiniaceae is a subfamily of Leguminosae according to Satish et al. [22].

### 2.2. Methods

#### 2.2.1. Extraction of Genomic DNA

For each accession, 0.1 g of fresh leaves from 14-day-old plants was finely ground in 750 *μ*l of ultrapure water using a mortar and pestle. The grindings from each sample collected in a 1.5 ml Eppendorf tube were centrifuged at 10,000 rotations per minute (RPM) for 10 min at 4°C. At the end of the centrifugation, the supernatant was removed, and 200 *μ*l of DNAzol was added to the pellet and homogenized. The tubes were then incubated at 65°C in a water bath for 2 h and then centrifuged for 15 min at 4°C. At the end of the centrifugation, the supernatant was collected and stored. The amount of DNA was determined using NanoDrop 2000. The DNA extraction took place in November 2020 in the Molecular Biology Unit of the Plant Genetics and Improvement Research Team of the Biosciences Laboratory of the Joseph KI-ZERBO University.

#### 2.2.2. PCR Amplification

For each DNA sample, a reaction volume of 25 *μ*l was prepared in an Eppendorf tube. The reaction mixture consisted of 4.5 *μ*l of 40 ng/*μ*l genomic DNA; 2 *μ*l of 10 *μ*M primer; 16 *μ*l of ultrapure water; and 2.5 *μ*l of the PCR Mix consisting of Taq polymerase (1U), dNTP (10 *μ*M), and buffer (10X) (10 mM Tris-HCL pH 9, 50 mM KCl, 1.5 mM MgCl_2_). After homogenization, amplification was performed in a thermal cycler following a program consisting of an initial DNA denaturation phase at 94°C for 3 minutes, followed by a series of 70 PCR cycles with denaturation at 94°C for 1 minute, hybridization at 48°C for 2 minutes, extension at 72°C for 2 minutes, and a final extension at 72°C for 15 minutes.

#### 2.2.3. Electrophoretic Migration and Band Reading

PCR products were subjected to electrophoretic migration on a 3% agarose gel prepared with a 1X TBE solution. A fluorescent developer, ethidium bromide (BET) 5%, was added to the agarose gel. Deposition of the amplification products was performed in the presence of a molecular weight marker ranging in size from 50 to 500 bp, and migration was performed at 90 V for 2 h in 0.5X TBE buffer. The reading of the amplification products was done under ultraviolet light from a transilluminator with a Canon EOS 1300D camera of 18 mega pixels. The bands were identified on the basis of their position on the gel. Thus, the molecular weight of each observed allele was noted for each accession and for each primer tested according to the weight marker.

PCR amplification, electrophoretic migration, and band reading were performed in the Laboratory of Plant Genetics and Biotechnology of the Department of Plant Production of the Institute of Environment and Agricultural Research (INERA).

#### 2.2.4. Data Analysis

Markers that gave clear bands were retained for data analysis. Genetic diversity within the *S. obtusifolia* population was analyzed at two levels: intrapopulation diversity and interpopulation diversity. Thus, the genetic parameters were calculated with the GenALEx software version 6.501 in order to evaluate the diversity of the whole collection and the diversity according to the climatic zones. A structure analysis was carried out using the DARwin V6.0 software. This software was first used to generate the dissimilarity matrix between accessions using the “simple matching” procedure. Then, from this dissimilarity matrix, a dendrogram was constructed using the neighbor-joining method. The ArcGIS 2012 software was used to construct a location map of survey sites and collections of accessions of *Senna obtusifolia* (L.).

*(1) Intrapopulation Diversity*. Several genetic parameters were estimated:

- The total number of alleles (At) or allelic richness which is the total number of alleles counted in a population. Its measure At = *n*−1, where *n* is the number of all the alleles provided by all the primers so that in a monomorphic population, its value is equal to 0;
- The number of different alleles per locus (Na) which is the sum of the alleles found per locus;
- The effective number of alleles (Ne) which is the inverse of the probability that, for a given locus, two randomly taken alleles are identical. Ae = 1/(1-*h*) = 1/Σpi2 where pi = the frequency of allele *i* of the locus considered and *h* = heterozygosity;
- The Shannon diversity index (*I*) was calculated according to the formula of [23] *I* = -ΣPi ln (Pi), where Pi is the frequency of allele *i*;
- The expected heterozygosity (He) and Nei's gene diversity index (*D*) which is the probability that at a given locus any two randomly selected alleles in the population are different. The expected heterozygosity rate (He) was calculated from the allele frequencies determined for each locus using the formula He = 1/*N* [n/n-1 (1-Σpi2)], where *N* is the number of loci, *n* is the number of accessions, and pi is the frequency of allele *i* of the locus considered.
- The polymorphism information content (PIC) which is a parameter that gives an estimate of the discriminatory power of a locus. It takes into account both the number of alleles expressed and the relative frequency of each allele [24]. It is calculated according to the formula PIC = 1-Σfi^2^, with fi being the frequency of each allele. The PIC varies from 0 for a monomorphic locus to 1 for a highly discriminating locus, with several alleles each in low and equal frequency.
- The polymorphism rate (*P*) which is the number of polymorphic loci (npj) divided by the total number of loci (*n* total). Its formula is *P* = npj/n total.

*(2) Interpopulation Diversity*. The accessions were subdivided into subpopulations according to the climatic zone factor. Genetic diversity parameters were estimated for each of these subpopulations. Two parameters were estimated in the description of the genetic diversity between the defined subpopulations. These are the indices of genetic differentiation between populations (Fst) and the minimum distance of Nei between pairs of genetic groups.

- The index of genetic differentiation between populations (Fst): its value is between 0 and 1. It is 0 in case of strong genetic similarity between subpopulations and 1 in case of fixation of different alleles in the subpopulations. Its formula is Fst = 1−HS/*H*_T_, where HS is the average of the expected heterozygosity of the subpopulations and *H*_T_ is the expected heterozygosity of the total population;
- The minimum distance of Nei between pairs of genetic groups: its value also varies from 0, for identical populations, to 1, for totally different populations.

## 3. Results

### 3.1. Level of Diversity of Microsatellite Markers Tested

**Figure 3:**
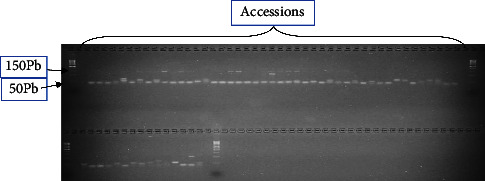
Migration profile obtained with the VM27 marker for 60 *Senna obtusifolia* (L.) accessions.

Among the eighteen (18) microsatellite markers tested, fifteen (15) were polymorphic (Figure 3). For the remaining three primers tested, two failed to obtain bands (VM36 and VM38) and one (VM39) yielded unreadable bands. Allele sizes ranged from 50 bp to 500 bp. Polymorphic markers revealed a total of 101 alleles (At), with an average of 6.733 alleles per locus. The number of different alleles per locus (Na) ranged from three (3) for primers VM35 and VM73 to fourteen (14) for primer VM31. The effective number of alleles (Ne) ranged from 1.11 for marker VM13 to 5.02 for marker VM72 with an average value of 2.33. With a mean value of 0.21, the observed heterozygosity ranged from 0.05 for markers VM22 and VM72 to 0.48 for marker VM31. As for the expected heterozygosity (He), it ranged from 0.10 for marker VM13 to 0.80 for marker VM72 with an average of 0.47. The Shannon diversity index (I) with a mean of 1.05 ranged from 0.25 for marker VM13 to 1.97 for marker VM31. The polymorphism information content (PIC) with a mean of 0.47 ranged from 0.10 for marker VM13 to 0.80 for marker VM68. The rate of polymorphic loci (P) was 100% for all markers (Table 2).

### 3.2. Interpopulation Diversity: Diversity according to the Climatic Zone Factor

The analysis of molecular variance (AMOVA) revealed a nonsignificant difference between accessions of the three climatic zones with a *P* value of 0.83. The factor climatic zone would therefore have a very weak influence on the expression of the genetic diversity of accessions (0%). The accessions factor plays a 100% role in the expression of this diversity (Table 3). Thus, in the Sudano-Sahelian zone, the number of alleles per locus (Na) was 6.07; the Shannon diversity index (I) was 1.04; and the polymorphism information content (PIC) was 0.48 compared to 3.37 (Na), 0.88 (I), and 0.43 (PIC), respectively, for the Sahelian zone. The values of these parameters are intermediate to those of the two previous zones in the Sudanian zone (Table 4).

The parameters, minimum distance of Nei (0.06) and differentiation index Fst (0.04), observed are higher between accessions from the Sahelian zone and those from the Sudanian zone. Low values of minimum Nei distance (0.03) and Fst differentiation index (0.02) were observed between accessions from the Sudan-Sahelian zone and those from the Sudanian zone (Table 5).

### 3.3. Structuring of Genetic Diversity

**Figure 4:**
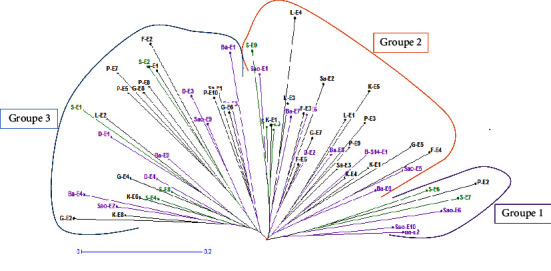
Dendrogram of the 60 accessions of *Senna obtusifolia* (L.) constructed using the neighbor-joining method. Green: Sahelian zone accessions; black: accessions from the Sudano-Sahelian zone; purple: accessions from the Sudanian zone.

The genetic diversity obtained was structured into three groups, independent of the climatic zone (Figure 4). The AMOVA analysis of this diversity shows a significant difference between these three groups at the 5% threshold (*P* value = 0.004). In the expression of genetic diversity, the intergroup genetic variance is 2% and the intragroup variance is 98% (Table 6), so the accession factor would intervene at 98% in the expression of this diversity.

- Group 1 with six (6) accessions includes three accessions from the Sudanian zone (Ba-E2, Sao-E6, and Sao-E10), one accession from the Sudano-Sahelian zone (P-E2), and two accessions from the Sahelian zone (S-E6 and S-E7);
- Group 2 with twenty-seven (27) accessions includes eight (8) accessions from the Sudanian zone (Ba-E6, Sao-E5, B-S14-E1, Ba-E8, D-E2, Ba-E5, Ba-E7, and Sao-E1), sixteen (16) accessions from the Sudano-Sahelian zone (F-E4, G-E5, K-E1, K-E4, Sa-E3, P-E9, P-E3, K-E5, L-E1, F-E5, G-E7, Sa-E2, F-E3, L-E3, L-E4, and F-E1), and three accessions from the Sahelian zone (S-E3, S-E5, and S-E9);
- Group 3 with twenty-seven (27) accessions includes nine (09) accessions from the Sudanian zone (Ba-E1, Ba-E3, Sao-E9, D-E3, Ba-E9, D-E1, D-E4, Ba-E4, and Sao-E2), fourteen (14) accessions from the Sudano-Sahelian zone (G-E6, Sa-E1, P-E10, F-E2, G-E1, P-E8, G-E8, P-E7, P-E5, L-E2, G-E4, K-E6, K-E8, and G-E2), and four accessions from the Sahelian zone (S-E2, S-E1, S-E8, and S-E4).

Genetic group 3 includes the majority of accessions that showed better agronomic performance during agromorphological characterization, i.e., seven accessions (G-E2, S-E8, G-E4, S-E1, S-E2, Sao-E9, and P-E10) out of 13.

In general, the genetic diversity parameters observed were high for group 3 and low for group 1 (Table 7). Thus, group 3 presented a Shannon diversity index (I) of 1.00 versus 0.63 for group 1, and the polymorphism information content (PIC) of group 3 was 0.47 versus 0.34 for group 1. Group 2 showed intermediate diversity parameters of the other two groups. The values of minimum Nei distance and Fst differentiation index were high between groups 1 and 3 (0.07 and 0.06) and low between groups 2 and 3 (0.03 and 0.02) (Table 8).

## 4. Discussion

Sixty (60) accessions of *Senna obtusifolia* were characterized using 18 SSR markers. Fifteen microsatellites were polymorphic (VM) markers and revealed one hundred and one (101) alleles in total, and the number of alleles per locus ranged from three (3) to fourteen (14) with an average of 6.73 alleles per locus. According to FAO [25], an average of at least four (4) distinct alleles per locus is required and denotes average genetic polymorphism. The average number of alleles in the present study (6.733) being higher than the average required by FAO [25], there would therefore be genetic polymorphism within the *Senna obtusifolia* collection. Work conducted by Mohanty et al. [16] revealed a total of 78 alleles for seven SSR markers of *Senna obtusifolia*. The difference between the results of the present study and previous work could be explained by the size and nature of the plant material used. According to Kalinowski [26], large samples generally contain more alleles than small samples. Furthermore, according to Ben Naceur et al. [27], the number of alleles per locus is influenced by several factors such as genotype and primer sequences as well as minor variations in the amplification protocol. Indeed, with the study [27] on the same VM markers, the number of alleles per locus varied from two to seven with an average of 4.7 in 90 cowpea accessions [19]. The low average number of alleles per locus observed for cowpea accessions could be explained by the nature of the accessions (90 cowpea lines) that originated from the International Institute of Tropical Agriculture breeding program and one wild cowpea and that their genetic base would therefore be relatively narrow. The rate of polymorphism being 100% for each of the markers would denote the ability of the markers to reveal the genetic diversity of the collection. Similar values were observed by Sawadogo [28] on forty-nine (49) accessions of *Solanum aethiopicum* L.

This very high rate of polymorphism testifies to the high level of polymorphism within the accessions and the efficiency of the markers used for the analysis of the genetic diversity of the populations studied [29]. According to Shete et al. [30], the expected heterozygosity and PIC are two values that can be used to determine the level of polymorphism of markers. The mean PIC value (0.47) in the present study was less than 0.5. According to Bostein et al. [31], markers with PIC greater than 0.5 are considered very highly informative. Eight (8) of the VM markers used have a PIC greater than 0.5 thus are very highly informative. However, it has been shown that PIC values between 0.25 and 0.5 provide reliable and usable information in characterization studies [32, 33].

Thus, there would be moderate variability within the *Senna obtusifolia* accessions in the present collection. The differentiation index Fst allowed the study of diversity between subpopulations. Thus, the genetic differentiation is weak between accessions of different climatic zones (average Fst of 0.03). According to Wright [34], Fst values indicate a genetic differentiation of the populations studied, weak if they are between 0 and 0.05, moderate between 0.05 and 0.15, important between 0.15 and 0.25, and very important if the value is higher than 0.25. The low value of the differentiation index Fst confirms the fact that the climatic zone factor plays a weak role in the expression of the genetic diversity of the species. Moreover, the AMOVA analysis revealed a nonsignificant difference (*P*=0.83) between accessions from different climatic zones. Thus, the genetic diversity of *Senna obtusifolia* would indeed be due to the nature of the plant material, as it is a plant that is always found in the wild. Indeed, according to Yuan et al. and Benor et al. [35, 36], domestication causes increased homogenization within cultivated plants in contrast to spontaneous accessions that possess significant genetic diversity.

According to Ould Ahmed et al. [29], total genetic diversity is the sum of intrapopulation genetic diversity and interpopulation diversity. Based on FST values, only 3% of the total variability would be due to differences between populations in different climatic zones.

The moderate genetic diversity in the present study could be due to the reproductive mode of *Senna obtusifolia*, which is cleistogamous, i.e., fertilization occurs before the opening of the flower buds. This phenomenon would reduce the genetic diversity within the species. The diversity of the sixty (60) accessions was structured into three distinct genetic groups. The accessions were grouped independent of their origins, thus forming composite groups. The accessions that presented better agronomic performances during agromorphological characterization were mostly found in the genetic group 3. The structuring of accessions into three genetic groups without taking into account their geographical origin confirms the low value of Fst (0.03), which reflects a weak influence of geographical origin on the total genetic diversity. Similar results were observed by Kiebre et al. [37, 38]. This would therefore suggest that geographic origin does not necessarily reflect the species characteristics and genetic diversity of *Senna obtusifolia* accessions in the present study. Similar results have been found by other authors such as Ambrosi et al. [39–41] on other species. This lack of correspondence between the molecular classification of accessions and geographic origin could reflect their introduction from the same origin. Similar results were reported by Konan and Mergeai [38].

With a mean Fst value of 0.06, genetic diversity was moderate among the three genetic groups. This difference was confirmed by a *P* value (0.004) significant at 5% in AMOVA analysis. Genetic diversity parameters were high for group 3 and low for group 1. In addition, this group contains the majority of the best agronomic performance accessions from the agromorphological evaluation. According to Nacambo et al. [11], an agromorphological evaluation carried out on these same accessions revealed a group of accessions (group 3) that showed better agronomic performance. The majority of the accessions in genetic group 3 are part of this group that showed better agronomic performance. Thus, the accessions of this group have a high number of primary ramifications and leaf biomass. They produce lots of long but slender fruits and lots of seeds. They flower early.

The results obtained in this study would therefore indicate the existence of a certain level of diversity that can be exploited by breeders.

## 5. Conclusion

This study revealed the existence of genetic diversity within the collection. The genetic diversity of *S. obtusifolia* accessions was structured into three groups independent of geographic origin. Group 3 consisting of twenty-seven (27) accessions had the highest genetic parameters, which also includes the majority of the accessions that have better agronomic performances.

## Figures and Tables

**Table 1:** SSR markers used to examine genetic variability among those used on *Vigna unguiculata* (cowpea).

SSR marker	Primer sequence	Repeat motif and number of repeats	Expected size (bp)
VM8	5′TGG GAT GCT GCA AAG ACA C	(AG) 16	285
	5′GAA AAC CGA TGC CAA ATA G		

VM13	5′CAC CCG TGA TTG CTT GTT G	(CT) 21	135
	5′GTC CCC TCC CTC CCA CTG		

VM14	5′AAT TCG TGG CAT AGT CAC AAG AGA	(AG) 24	144
	5′ATA AAG GAG GGC ATA GGG AGG TAT		

VM21	5′TAG CAA CTG TCT AAG CCT CA	(AT) 17	179
	5′CCA ACT TAA CCA TCA CTC AC		

VM22	5′GCG GGT AGT GTA TAC AAT TTG	(AG) 12	217
	5′GTA CTG TTC CAT GGA AGA TCT		

VM27	5′GTC CAA AGC AAA TGA GTC AA	(AAT) 5. (TC) 14. (AC) 3	207
	5′TGA ATG ACA ATG AGG GTG C		

VM31	5′CGC TCT TCG TTG ATG GTT ATG	(CT) 16	200
	5′GTG TTC TAG AGG GTG TGA TGG TA		

VM32	5′GAA AAA GGG AGG AAC AAG CAC AAC	(AG) 10	177
	5′AGC GAA AAC ACG GAA CTG AAA TC		

VM35	5′GGT CAA TAG AAT AAT GGA AAG TGT	(AG) 11. (T) 9	127
	5′ATG GCT GAA ATA GGT GTC TGA		

VM36	5′ACT TTC TGT TTT ACT CGA CAA CTC	(CT) 13	160
	5′GTC GCT GGG GGT GGC TTA TT		

VM38	5′AAT GGG AAA AGA AAG GGA AGC	(AG) 10. (AC) 5	135
	5′TCG TGG CAT GCA GTG TCA G		

VM39	5′GAT GGT TGT AAT GGG AGA GTC	(AC) 13. (AT) 5. (TACA) 4	212
	5′AAA AGG ATG AAA TTA GGA GAG CA		

VM68	5′CAA GGC ATG GAA AGA AGT AAG AT	(GA) 15	254
	5′TCG AAG CAA CAA ATG GTC ACA C		

VM69	5′CAA AGC ATT GGG CCC TTG T	(AG) 19	217
	5′GGC TTT GGG ACC TCC TTT CC		

VM70	5′AAA ATC GGG GAA GGA AAC C	(AG) 20	186
	5′GAA GGC AAA ATA CAT GGA GTC AC		

VM71	5′TCG TGG CAG AGA ATC AAA GAC AC	(AG) 12. (AAAG) 3	225
	5′TGG GTG GAG GCA AAA ACA AAA C		

VM72	5′TGC TGA AGT GAA CAA TCG C	(AG) 20	310
	5′CCT TCT CCA ACA ACT CTA C		

VM73	5′CGG CGT GAT TTG GGG AAG AAG	(AG) 15	201
	5′CTA GTA ACG GCC GCC AGT GTC CTG		

**Table 2:** Diversity parameters of the 15 polymorphic markers.

Primers	Parameters							
	N	Na	Ne	I	Ho	He	PIC	P (%)
VM8	60	9.00	3.45	1.55	0.50	0.71	0.702	100
VM13	60	4.00	1.11	0.25	0.08	0.10	0.10	100
VM14	60	12.00	2.53	1.47	0.40	0.61	0.61	100
VM21	60	8.00	2.38	1.32	0.33	0.58	0.59	100
VM22	60	6.00	1.53	0.79	0.05	0.35	0.21	100
VM27	60	6.00	2.01	0.98	0.10	0.50	0.50	100
VM31	60	14.00	4.78	1.97	0.48	0.79	0.79	100
VM32	60	6.00	1.74	0.98	0.28	0.43	0.39	100
VN35	60	3.00	1.17	0.33	0.12	0.14	0.11	100
VM68	60	9.00	2.26	1.34	0.10	0.56	0.80	100
VM69	60	4.00	2.60	1.08	0.25	0.62	0.61	100
VM70	60	4.00	1.32	0.53	0.13	0.24	0.24	100
VM71	60	5.00	1.86	0.98	0.13	0.46	0.43	100
VM72	60	8.000	5.02	1.80	0.05	0.80	0.76	100
VM73	60	3.000	1.23	0.38	0.17	0.18	0.18	100
Mean	60	6.733	2.33	1.05	0.21	0.47	0.47	100

N: number of accessions; Na: number of different alleles per locus in the population; Ne: number of effective alleles; I: Shannon diversity index; Ho: observed heterozygosity; He: expected heterozygosity; PIC: polymorphism information content; P: polymorphic loci rate.

**Table 3:** Analysis of molecular variance (AMOVA) of accessions from the three climatic zones.

Source	Df	SS	MS	Est. Var.	D (%)
Variance between climatic zones	2	6.71	3.36	0.00	0
Variance within climatic zones	57	212.99	3.74	3.74	100
Total	59	219.70		3.74	100

Df: degree of freedom; SS: sum of squares; MS: mean square; Est. Var.: estimated variance; D: total variance distribution.

**Table 4:** Genetic diversity parameters of accessions from the 3 climatic zones.

Parameters	Climatic zones							
	N	Na	Ne	I	Ho	He	PIC	P (%)
Sahelian zone	9.00	3.73	2.14	0.88	0.22	0.46	0.43	93.33
Sudanese-Sahelian zone	31.00	6.07	2.48	1.04	0.21	0.48	0.48	100
Sudanese zone	20.00	5.00	2.11	0.91	0.21	0.43	0.47	93.33
Average	20.00	4.93	2.25	0.94	0.21	0.46	0.46	95.56

N: number of accessions; Na: number of different alleles per locus in the population; Ne: number of effective alleles; I: Shannon diversity index; He: expected heterozygosity; PIC: polymorphism information content; P: polymorphic loci rate.

**Table 5:** Genetic differentiation between accessions from the three climatic zones.

Climatic zones	Parameters					
	Minimum distance of Nei			Index of genetic differentiation Fst		
	SZ	SSZ	SUZ	SZ	SSZ	SUZ
Sahelian zone	0.00			0.00		
Sudanese-Sahelian zone	0.03	0.00		0.02	0.00	
Sudanese zone	0.06	0.03	0.00	0.04	0.02	0.00

SZ: Sahelian zone; SSZ: Sudanese-Sahelian zone; SUZ: Sudanese zone.

**Table 6:** Analysis of molecular variance (AMOVA) of accessions from the three genetic groups with *P* value obtained after 999 permutations.

Source	Df	SS	MS	Est. Var.	D (%)
Variance between groups	2	10.34	5.17	0.09	2
Variance in groups	57	208.15	3.65	3.65	98
Total	59	218.48		3.74	100

Df: degree of freedom; SS: sum of squares; MS: mean square; Est. Var.: estimated variance; D: total variance distribution.

**Table 7:** Genetic diversity parameters of *Senna obtusifolia* according to genetic groups.

Populations	Parameters							
	N	Na	Ne	I	Ho	He	PIC	P (%)
Pop 1	6.00	2.67	1.93	0.63	0.14	0.34	0.34	73.33
Pop 2	27.00	5.33	2.23	0.95	0.24	0.46	0.46	100
Pop 3	27.00	5.53	2.34	1.00	0.20	0.47	0.47	100
Average	20.00	4.51	2.16	0.86	0.20	0.42	0.42	91.11

N: number of accessions; Na: number of different alleles per locus in the population; Ne: number of effective alleles; I: Shannon diversity index; He: expected heterozygosity; PIC: polymorphism information content; P: polymorphic loci rate.

**Table 8:** Genetic differentiation of the three genetic groups.

Populations	Parameters					
	Minimum distance of Nei			Index of genetic differentiation Fst		
	Pop 1	Pop 2	Pop 3	Pop 1	Pop 2	Pop 3
Pop 1	0.00			0.00		
Pop 2	0.03	0.00		0.05	0.00	
Pop 3	0.07	0.03	0.00	0.06	0.02	0.00

Pop 1: genetic group 1; Pop 2: genetic group 2; Pop 3: genetic group 3.

## Data Availability

The data used to support the findings of this study are available from the corresponding author upon request.

## Supplementary Materials

Supplementary Materials See the appendix for additional data.
