# Supplementary material for: Assessment of Genetic Diversity of a Collection of Senna obtusifolia (L.) Irwin and Barneby Using SSRs Markers in Burkina Faso
Source: ScientificWorldJournal. 2023 Jun 5;2023:3761799. doi: 10.1155/2023/3761799 (PMC10260308; doi:10.1155/2023/3761799)
Supplement: Supplementary Materials — See the appendix for additional data. [file 3761799.f1.docx]

530**Appendix**: lists and meanings of acronyms of accessions

| **Acronyms and meaningsSigles** | |
| --- | --- |
| B-S14-E1 : Bobo secteur 14 échantillon 1  Ba-E1 : Banakélédaga sample 1  Ba-E2 : Banakélédaga sample 2  Ba-E3 : Banakélédaga sample 3  Ba-E4 : Banakélédaga sample 4  Ba-E7 : Banakélédaga sample 7  Ba-E5 : Banakélédaga sample 5  Ba-E6 : Banakélédaga sample 6  Ba-E8 : Banakélédaga sample 8  Ba-E9 : Banakélédaga sample9  D-E3 : Darsalamy sample3  D-E4 : Darsalamy sample 4  D-E1 : Darsalamy sample 1  D-E2 : Darsalamy sample 2  F-E4 : Fakouna sample 4   F-E1 : Fakouna sample 1   F-E2 : Fakouna sample 2   F-E3 : Fakouna sample 3    F-E5 : Fakouna sample5  G-E8 : Garghin sample8  G-E2 : Garghin sample 2  G-E4 : Garghin sample4   G-E7 : Garghin sample 7    G-E1 : Garghin sample 1  G-E5 : Garghin sample 5  G-E6 : Garghin sample 6   K-E1 : Koudmi sample 1  K-E4 : Koudmi sample 4  K-E5 : Koudmi sample 5 | K-E6 : Koudmi sample6  K-E8 : Koudmi sample 8  L-E2 : Lonkakuy sample2  L-E3 : Lonkakuy sample 3  L-E4 : Lonkakuy sample 4   L-E1 : Lonkakuy sample 1  P-E10 : Bassem-poessin sample 10  P-E3 : Bassem-poessin sample 3  P-E9 : Bassem-poessin sample 9  P-E2 : Bassem-poessin sample 2  P-E5 : Bassem-poessin sample 5   P-E7 : Bassem-poessin sample 7   P-E8 : Bassem-poessin sample 8  S-E1 : Sissamba sample 1  S-E2 : Sissamba sample 2  S-E7 : Sissamba sample 7  S-E8 : Sissamba sample 8  S-E6 : Sissamba sample 6   S-E9 : Sissamba sample9  S-E3 : Sissamba sample 9  S-E4 : Sissamba sample 4   S-E5 : Sissamba échantillon 5  Sa-E3 : Saaba sample3  Sa-E1 : Saaba sample 1  Sa-E2 : Saaba é sample 2  Sao-E1 : Saouileni sample 1  Sao-E9 : Saouileni sample 9  Sao-E10 : Saouileni sample 10  Sao-E5 : Saouileni sample 5   Sao-E6 : Saouileni sample 6 |
